# Supplementary material for: Seq-ing the SINEs of central nervous system tumors in cerebrospinal fluid
Source: Cell Rep Med. 2023 Aug 7;4(8):101148. doi: 10.1016/j.xcrm.2023.101148 (PMC10439243; doi:10.1016/j.xcrm.2023.101148)
Supplement: Document S1. Figure S1 [file mmc1.pdf]

Cell Reports Medicine, Volume *4*

Supplemental information

Seq-ing the SINEs of central nervous system tumors in cerebrospinal fluid

Christopher Douville, Samuel Curtis, Mahmoud Summers, Tej D. Azad, Jordina Rincon-Torroella, Yuxuan Wang, Austin Mattox, Bracha Avigdor, Jonathan Dudley, Joshua Materi, Divyaansh Raj, Sumil Nair, Debarati Bhanja, Kyle Tuohy, Lisa Dobbyn, Maria Popoli, Janine Ptak, Nadine Nehme, Natalie Silliman, Cherie Blair, Kathy Judge, Gary L. Gallia, Mari Groves, Christopher M. Jackson, Eric M. Jackson, John Laterra, Michael Lim, Debraj Mukherjee, Jon Weingart, Jarushka Naidoo, Carl Koschmann, Natalya Smith, Karisa C. Schreck, Carlos A. Pardo, Michael Glantz, Matthias Holdhoff, Kenneth W. Kinzler, Nickolas Papadopoulos, Bert Vogelstein, and Chetan Bettegowda

Supplemental Information:

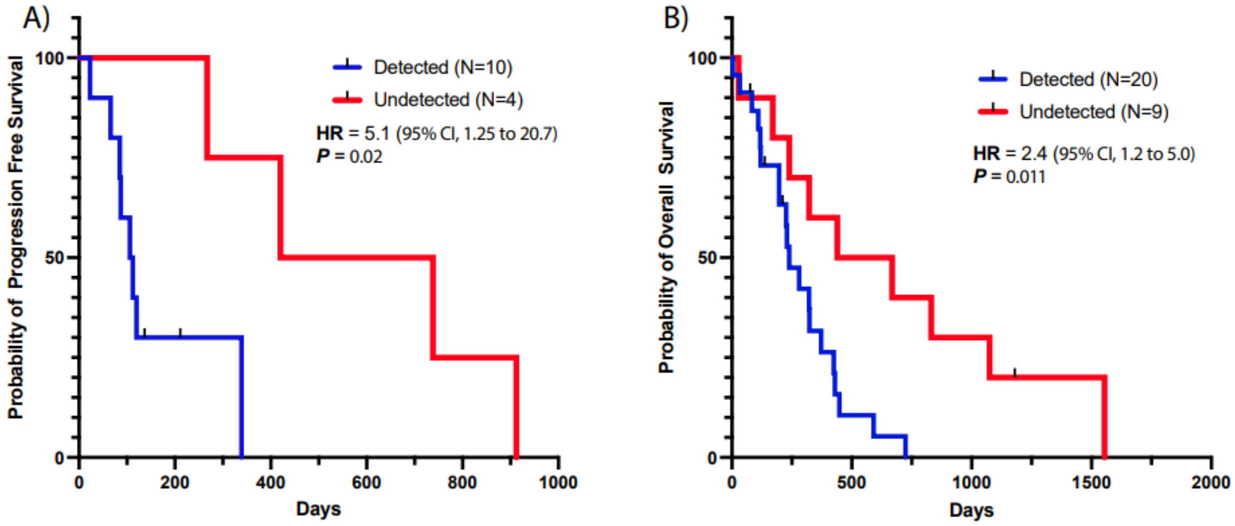

Supplemental Figure S1: Figure S1: Survival Analysis of new diagnosed GBM patients based on detectable copy number alterations in the CSF, related to STAR Methods. A) Progression Free Survival (PFS) B) Overall Survival (OS).

Supplemental Table S1: A Full Description of the Sample Demographics in the study, related to STAR Methods.

Supplemental Table S2: Genomic Coordinates for the Focal Regions Evaluated, related to Figure 1.

Supplemental Table S3: Real-CSF Raw Predictive Features Listed for all Manuscript Samples, related to STAR Methods.

Supplemental Table S4: Real-CSF Calls For Matched Plasma Samples, related to STAR Methods.
