# Supplementary material for: Seq-ing the SINEs of central nervous system tumors in cerebrospinal fluid
Source: Cell Rep Med. 2023 Aug 7;4(8):101148. doi: 10.1016/j.xcrm.2023.101148 (PMC10439243; doi:10.1016/j.xcrm.2023.101148)
Supplement: Document S2. Article plus supplemental information [file mmc6.pdf]

Cell Reports
Medicine

Report

Seq-ing the SINEs of central nervous system tumors in cerebrospinal fluid

Graphical abstract

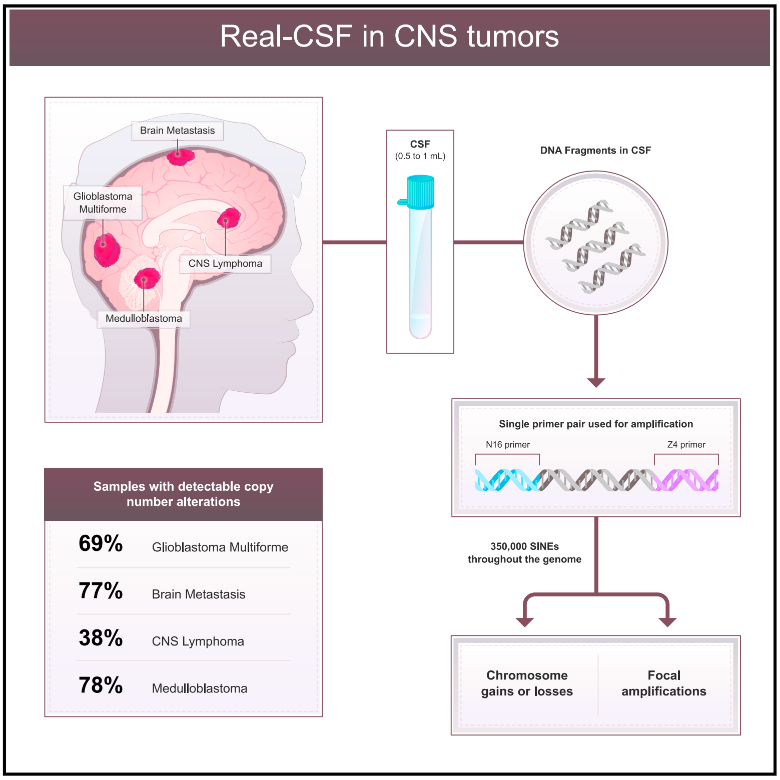

Highlights

- Limited DNA in the cerebrospinal fluid (CSF) poses challenges for genetic techniques
- Copy-number alterations are a feature of nearly all CNS cancers
- Real-CSF works in limited starting material and identifies copy-number alterations
- Real-CSF can detect neoplasia in CSF across a heterogeneous set of CNS cancers

Authors

Christopher Douville, Samuel Curtis, Mahmoud Summers, ..., Nickolas Papadopoulos, Bert Vogelstein, Chetan Bettegowda

Correspondence

cbetteg1@jhmi.edu

In brief

Douville et al. introduce Real-CSF—a molecular technique designed to identify copy-number alterations spread throughout the genome from limited starting material in cerebrospinal fluid. Even though central nervous system cancers comprise a heterogeneous set of tumors, nearly all will contain copy-number alterations, making this a putative multi-cancer biomarker.

Douville et al., 2023, Cell Reports Medicine *4*, 101148
August 15, 2023 © 2023 The Author(s).
https://doi.org/10.1016/j.xcrm.2023.101148

CellPress

Cell Reports Medicine

CellPress
OPEN ACCESS

Report

Seq-ing the SINEs of central nervous system tumors in cerebrospinal fluid

Christopher Douville,[1,2,3,4] Samuel Curtis,[1,2,3,4] Mahmoud Summers,[1,2,3,4] Tej D. Azad,[5] Jordina Rincon-Torroella,[1,2,3,4,5] Yuxuan Wang,[1,2,3,4] Austin Mattox,[1,2,3,4] Bracha Avigdor,[1,2,3,4] Jonathan Dudley,[1,2,3,4,6] Joshua Materi,[5] Divyaansh Raj,[5] Sumil Nair,[5] Debarati Bhanja,[7] Kyle Tuohy,[7] Lisa Dobbyn,[1,2,3,4] Maria Popoli,[1,2,3,4] Janine Ptak,[1,2,3,4] Nadine Nehme,[1,2,3,4] Natalie Silliman,[1,2,3,4] Cherie Blair,[1,2,3,4] Kathy Judge,[1,2,3,4] Gary L. Gallia,[5] Mari Groves,[5] Christopher M. Jackson,[5] Eric M. Jackson,[5] John Laterra,[8] Michael Lim,[9] Debraj Mukherjee,[5] Jon Weingart,[5] Jarushka Naidoo,[10] Carl Koschmann,[11] Natalya Smith,[7] Karisa C. Schreck,[1,8] Carlos A. Pardo,[8] Michael Glantz,[7] Matthias Holdhoff,[1] Kenneth W. Kinzler,[1,2,3,4] Nickolas Papadopoulos,[1,2,3,4] Bert Vogelstein,[1,2,3,4,12] and Chetan Bettegowda[1,2,3,4,5,13,*]
[1]Department of Oncology, The Sidney Kimmel Cancer Center, Johns Hopkins University School of Medicine, 733 N. Broadway, Baltimore, MD 21205, USA
[2]The Ludwig Center, Johns Hopkins University School of Medicine, 733 N. Broadway, Baltimore, MD 21205, USA
[3]The Sol Goldman Pancreatic Cancer Research Center, Johns Hopkins University School of Medicine, 733 N. Broadway, Baltimore, MD 21205, USA
[4]Sidney Kimmel Comprehensive Cancer Center, Johns Hopkins University School of Medicine, Baltimore, MD 21287, USA
[5]Department of Neurosurgery, Johns Hopkins University School of Medicine, 733 N. Broadway, Baltimore, MD 21205, USA
[6]Department of Pathology, Johns Hopkins University School of Medicine, 733 N. Broadway, Baltimore, MD 21205, USA
[7]Department of Neurosurgery, Pennsylvania State University, Hershey, PA, USA
[8]Department of Neurology, Johns Hopkins Medical Institutions, Baltimore, MD, USA
[9]Department of Neurosurgery, Stanford University, Palo Alto, CA, USA
[10]Department of Oncology, Beaumont Hospital, Dublin, Ireland
[11]Division of Pediatric Oncology, University of Michigan, Ann Arbor, MI, USA
[12]The Howard Hughes Medical Institute, Johns Hopkins University School of Medicine, 733 N. Broadway, Baltimore, MD 21205, USA
[13]Lead contact
*Correspondence: cbetteg1@jhmi.edu
https://doi.org/10.1016/j.xcrm.2023.101148

SUMMARY

It is often challenging to distinguish cancerous from non-cancerous lesions in the brain using conventional diagnostic approaches. We introduce an analytic technique called Real-CSF (repetitive element aneuploidy sequencing in CSF) to detect cancers of the central nervous system from evaluation of DNA in the cerebrospinal fluid (CSF). Short interspersed nuclear elements (SINEs) are PCR amplified with a single primer pair, and the PCR products are evaluated by next-generation sequencing. Real-CSF assesses genome-wide copy-number alterations as well as focal amplifications of selected oncogenes. Real-CSF was applied to 280 CSF samples and correctly identified 67% of 184 cancerous and 96% of 96 non-cancerous brain lesions. CSF analysis was considerably more sensitive than standard-of-care cytology and plasma cell-free DNA analysis in the same patients. Real-CSF therefore has the capacity to be used in combination with other clinical, radiologic, and laboratory-based data to inform the diagnosis and management of patients with suspected cancers of the brain.

INTRODUCTION

Central nervous system (CNS) neoplasms comprise a heterogeneous class of tumors that are either primary, i.e., originate in the brain or the spinal cord, or metastatic, i.e., cancers that spread to the CNS from another organ. Approximately 24,500 cases of primary brain cancers occur a year in the United States, with the most common being glioblastoma in adults and medulloblastoma in children.[1] Metastatic spread to the brain is even more common, accounting for 100,000 cases a year in the United States, with lung and breast being the most frequent. Cancers can spread to the brain matter itself, known as parenchymal metastases (PMs), or to the covering of the brain, known as leptomeningeal disease (LMD).

The small number of reliable, quantitative biomarkers for the diagnosis and monitoring of cancers in the CNS poses challenges for diagnosis of patients with suspected brain cancers. The current gold standard is cytology of cerebrospinal fluid (CSF), which has a sensitivity that ranges from 2% to 50% depending on the cancer type.[2] To achieve maximum sensitivity, cytology requires large (>10 mL) volumes of CSF, sometimes necessitating serial lumbar punctures.[3] Magnetic resonance and other imaging procedures cannot reliably distinguish cancer from inflammatory or other non-neoplastic processes.[4]

This is an open access article under the CC BY license (http://creativecommons.org/licenses/by/4.0/).

Therefore, biopsy remains the only means for definitive diagnosis of CNS neoplasms. Brain biopsies require general anesthesia and hospitalization, are fraught with risks including neurological injury, are associated with substantial financial burden, and, in up to 15% of cases, still do not yield a definitive diagnosis.[5–8]

There have been several promising types of biomarkers proposed to identify cancers of the CNS. Given the relative lack of brain-derived analytes in the blood,[9–12] CSF has become an appealing biofluid for diagnosis.[13–19] While CSF sampling is more invasive than venipuncture, it is already part of the standard of care for the diagnosis or management of several types of CNS disease. For example, in lymphomas, CSF is routinely obtained for cytologic and flow cytometric tests. Though the sensitivity of cytology for CNS cancers is relatively low, its specificity is high,[2,20] and positive cytology results can be useful for patient management. For example, patients with lymphoma with CSF-positive cytology can be advised to proceed directly to chemotherapy and radiation without biopsy, with a positive impact on survival and quality of life.[21,22]

In the current study, we describe our efforts to develop a simple strategy, called Real-CSF (repetitive element aneuploidy sequencing in CSF), for the evaluation of several of the most common and debilitating forms of brain cancers: glioblastomas, metastatic lesions, lymphomas, and medulloblastomas. These assays were performed on DNA purified from 1 mL CSF, including all cells and cell-free fluid within the CSF aliquot, and evaluated chromosome abnormalities. We also compared whether cell-free DNA from plasma could as easily detect such chromosome abnormalities as CSF.

RESULTS

Patient characteristics

Two independent cohorts of patients were evaluated in this study: a training set and a validation set. The training set was composed of CSF samples from 85 patients: 31 with glioblastoma (GBM), 13 with metastasis from primary tumors outside the brain, 7 with lymphoma, and 34 without cancer. The validation set was composed of CSF samples from 195 patients: 27 with GBM (five of which were pediatric H3K27M diffuse midline gliomas), 52 with metastasis from primary tumors outside the brain, 27 with CNS lymphoma, 23 with medulloblastoma, and 62 without cancer. Information with respect to the timing of the CSF sample collection (prior to diagnosis or during management of patients with known cancers) is recorded in Table S1. Thirteen metastatic samples were previously analyzed and reported in Naidoo et al.[23] The CSF was obtained in almost all cases from lumbar puncture or aspiration from a ventricular catheter placed as part of standard of care. The demographics of the training and validation sets are presented in Table S1.

Rationale and background of the assay

CNS neoplasms comprise a heterogeneous class of tumors and an equally diverse landscape of genetic alterations. Identifying the optimal combination of genetic markers that could encompass all CNS cancers is difficult. There is often insufficient starting material in CSF to query all somatic mutations and translocations across all potential driver genes. Aneuploidy or the presence of an abnormal number of chromosomes is a feature of most CNS cancer cells.[24] Nearly all GBM, medulloblastoma, and metastatic cancers are aneuploid.[24–26] CNS lymphoma has a notably lower rate of aneuploidy but still occurs in the majority of these cancers (71%).[24] We hypothesize that aneuploidy could act as a viable biomarker for CNS cancers, with variation in performance based on the prevalence of copy-number changes.

Here, we evaluate aneuploidy as a potential biomarker with a simple PCR assay that uses a single primer pair to amplify ~350,000 short interspersed nuclear elements (SINEs) throughout the genome.[27] The PCR products can then be assessed by massive parallel sequencing to identify chromosomal gains and losses as well as focal amplifications and deletions. The efficiency of PCR copying DNA is high (>90%) and has even been able to reliably detect aneuploidy in as little as a few pg DNA—representing half of a diploid cell.[27] Given the limited starting material in CSF, this assay is well suited to evaluate aneuploidy as a possible CNS biomarker.[28] We named this approach Real-CSF.

Training set data

We used the training set to optimize the machine-learning algorithms and other aspects of the analytic workflow. We first assessed whether the presence of large-scale chromosome arm gains and losses (aneuploidy) could detect cancerous lesions with high specificity. To assess the degree of aneuploidy, Z_w scores for each of the 39 non-acrocentric chromosome arms in each sample were calculated (detailed in the STAR Methods). These chromosome arm-level Z_w scores were then integrated into a single score, called the global aneuploidy score. The global aneuploidy score reflects the likelihood that a sample has gained or lost at least one chromosome, with the magnitude of the score reflecting both the number of chromosome arms that were altered as well as the fraction of cells in the CSF in which these changes occurred.

Based on cross-validation in the training set, we established a global aneuploidy score threshold of 0.25 for subsequent validation. This threshold correctly identified 63% (95% confidence interval [CI], 48%–75%) of the 85 CSF samples from patients with cancer—58% of patients with GBM, 92% of patients with metastases to the brain, and 29% of patients with lymphomas. Of the 34 patients with brain lesions but without cancer, none had global aneuploidy scores <0.25, yielding a specificity of 100% (95% CI, 90%–100%).

We next sought to determine whether the evaluation of focal amplifications of oncogenes, i.e., those involving only a small region surrounding an oncogene rather than the entire chromosome arm on which the oncogene is located, could detect other CNS cancers using data generated with RealSeqS (STAR Methods). For this analysis, we first selected oncogenes that were relatively frequently amplified in CNS cancers based on data from The Cancer Genome Atlas (TCGA).[29] Using the training cohort to assess the potential value of these genes, we narrowed the list to four genes—*MDM4*, *EGFR*, *CDK4*, and *HER2*, with genomic coordinates listed in Table S2.

For each of these four genes, we calculated a focal amplification score and a threshold for positivity in an analogous way to

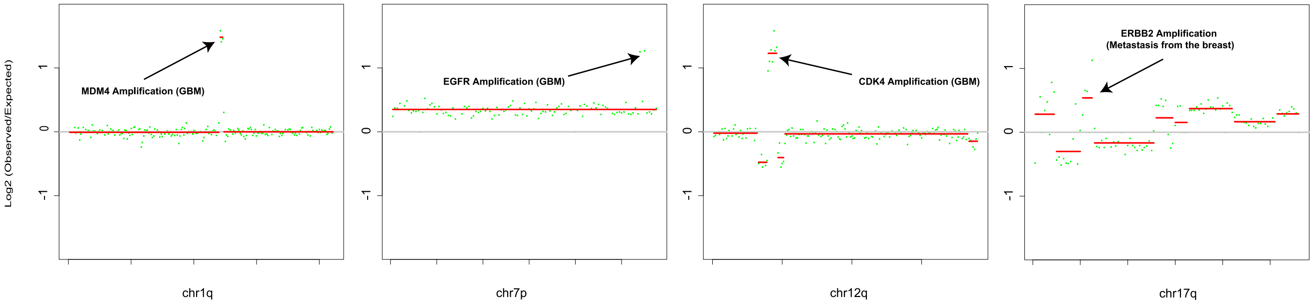

Figure 1. Representative focal changes used in Real-CSF

The Real-CSF focal panel calls focal changes surrounding the following genes: (A) 1.5 M focal amplification of MDM4 at 1q32.1 (chr1: 203,800,000–205,300,000 hg19); (B) 3.5 MB focal amplification of CDK4 at 12q14.1 (chr12: 57,600,000–61,100,000 hg19); (C) 1.5 MB focal amplification of EGFR at 7p11.2 (chr7: 54,200,000–55,700,000 hg19); and (D) 2.5 MB focal amplification of ERBB2 17q12 (chr17: 35,300,000–37,800,000 hg19)

that described above for the global aneuploidy score. We found that 31% (95% CI, 20%–46%) of the 85 CSF samples from patients with CNS cancers scored positively (examples in Figure 1). Using a Boolean OR gate, we defined a sample as positive in Real-CSF if it scored positively either for global aneuploidy or for a focal amplification of any of the four genes. Two-thirds (67%; 95% CI, 52%–79%) of the samples from patients with cancers scored positively in this composite Real-CSF assay, including 65% of the patients with GBM, 92% of the patients with metastatic lesions to the brain, 29% of the patients with lymphomas, and no patients without a CNS cancer (Table S3).

Validation set

The validation set provided an opportunity to independently assess the sensitivity and specificity of Real-CSF. Importantly, the validation set included samples from four different institutions, while samples in the training set were all from only one of these four institutions. This multi-institutional acquisition was intentionally designed to minimize confounders that can be observed when a classification method based on samples from a single institution is applied to samples from other institutions.[30] The validation set also included patients with medulloblastoma, a tumor type not represented in the training set but expected to exhibit aneuploidy as well as focal amplifications.

Using the thresholds pre-defined by the training set data, 68% of the patients with cancer scored positively (95% CI, 59%–76%). This included 74% of patients with GBM, 73% of patients with metastatic lesions, 41% of patients with lymphomas, and 78% of patients with medulloblastomas (Table 1). Of the 62 samples from patients without CNS cancers in the validation set, four (6.4%; 95% CI, 5.6%–12%) scored positively in Real-CSF. No sample type present in both the training and the validation sets had statistically different detection rates ($p > 0.05$ two-proportion Z-test).

Survival analysis

There were sufficient follow-up data to analyze progression-free survival (PFS) and overall survival (OS) in subjects with GBM treated at one of the institutions, JHU. Of the 14 patients with newly diagnosed GBM, 10 had detectable levels of CSF-tDNA, while 4 did not. The individuals with detectable levels of CSF-tDNA had an OR of 5.1 ($p = 0.02$, log-rank test; Figure S1A) for disease progression when compared with those without CSF-tDNA detection. Of the 29 patients with newly diagnosed and recurrent GBM, 20 had detectable levels of CSF-tDNA, and 9 had undetectable levels. The cases with detectable CSF-tDNA had an OR of 2.4 for poorer OS ($p = 0.011$, log-rank test; Figure S1B). The average survival for individuals with IDH mutant GBM is three times as long as for individuals with IDH wild-type GBM.[31] Only 2 samples were IDH mutant, and both were Real-CSF positive. The fact these and other individuals that were positive had a poorer OS suggests that Real-CSF is detecting biological behavior.

Concordance with whole-genome sequencing

To orthogonally validate the copy-number alterations identified by Real-CSF, we performed conventional whole-genome sequencing (WGS) on the CSF DNA from 43 patients with CNS cancers and from 28 without cancer. The sequencing depth averaged ~34.3 M read pairs, and copy-number alterations were identified with WisecondorX (see STAR Methods).[32] Among the 43 cancer samples, Real-CSF identified 106 chromosome arm-level gains ($Z > 7.5$) and 126 losses ($Z < -7.5$). Nearly all of these gains (96%) and losses (90%) were identified with WGS. The majority of the chromosome arms gains or losses (9 of 17) that were identified with Real-CSF, but not with WGS, had Z scores ($Z > 5$ or < -5) just below the Z score of [7.5] required for positivity. Of the 28 CSF DNA samples from patients without cancer, 1,091 of 1,092 of the chromosome arms evaluated (39 arms × 28 patients) were identified as euploid by WGS. The one arm that was aneuploid in one patient was chromosome (chrom) 19p, which has been reported to have a relatively high false positive rate with WGS.[32] Notably, Real-CSF scored all 1,092 chromosome arms as euploid.

Comparison with cytology

Of the 121 patients with cancer from either the training or validation sets in whom cytology was available, only 28 (23%; 95% CI, 16%–32%) were detectable by cytology. The sensitivity of Real-CSF in the same 121 patients was 69%, considerably higher than that of cytology (Figure 2B; $p < 2.2e{-16}$ binomial proportions test). However, not all patients who had positive cytology also scored positively with Real-CSF, or vice versa (Figure 3B).

Table 1. Summary of patient cohorts and performance metrics

	Non-cancer		GBM		Metastasis		Lymphoma		Medulloblastoma	
	Specificity (%)	n	Sensitivity (%)	n	Sensitivity (%)	n	Sensitivity (%)	n	Sensitivity (%)	n
Training	100	34	65	31	92	13	29	7	N/A	0
Validation	94	62	74	31	73	52	41	27	78	23

Together, either Real-CSF or cytology was positive in 73% (95% CI, 64%–80%) of cases.

Analysis of plasma from patients with CNS cancers

Given that plasma is much more easily accessible than CSF, it was of interest to determine whether plasma could substitute for CSF in RealSeqS assays for aneuploidy or focal amplifications. We have previously described STAR Methods to compute global aneuploidy and focal amplification scores in cell-free DNA (cfDNA) from plasma from individuals without cancer and in patients with cancers of organs other than the brain. In the current study, we evaluated plasma in 65 patients with CNS cancers (GBM, lymphoma, or medulloblastoma; Table S4). We also evaluated plasma samples from 185 non-cancer individuals (trigeminal neuralgia, hydrocephalus, and neurodegenerative diseases) to assess specificity. Positive global aneuploidy scores were obtained in nine of the 65 patients with cancer (sensitivity of 14%; 95% CI, 6.9%–25%) and in two of the 185 controls (specificity of 98.9%; 95% CI, 96%–100%). No focal amplifications were observed in the plasma of patients with or without cancer.

Thirty-five of the 65 patients with brain cancer who donated plasma had also donated CSF (Table S4). In these matched samples, 66% (95% CI, 48%–81%) of the CSF samples scored as positive, while 23% (95% CI, 10%–40%) of the plasma samples scored as positive. Five patients scored positively in both plasma and CSF (Figure 2C). Eighteen of the 35 patients scored positively in CSF but not in plasma, and conversely, three patients scored positively in plasma but not CSF (Figure 3C). Thus, at similar specificities, CSF DNA was a more sensitive analyte than plasma cfDNA for the detection of chromosomal alterations ($p < 0.00001$, Z score for 2 population proportions).

DISCUSSION

Biomarkers for distinguishing non-neoplastic from neoplastic lesions can help resolve the diagnostic conundrum that is posed for the thousands of patients with space-occupying lesions of the brain each year. There are many types of non-neoplastic processes that can present with an imaging abnormality that mimics a brain tumor. Correctly identifying these abnormalities as non-malignant is important because such patients rarely benefit from surgical biopsy and can almost always be managed non-operatively, and a diagnosis other than CNS malignancy can alleviate much of the anxiety experienced by patients without cancer after imaging. In contrast, patients with cancer could benefit from correct diagnosis by spurring immediate consultation with appropriate neurosurgical and oncology specialists.

The ideal biomarker would not require brain tissue, would require only a small amount of CSF, would be simple to interpret, would be relatively inexpensive, could identify a myriad of cancer types, and could be readily automated. Real-CSF satisfies all of these criteria and appears to be more informative than cytology, the current gold standard. Previous studies have demonstrated that low-pass WGS can identify chromosomal copy-number alterations in the CSF from individuals with select brain cancers.[13,14,33] WGS has some advantages over Real-CSF, as it can detect translocations as well as other genetic events that do not involve SINE elements. Conversely, Real-CSF has distinct advantages over WGS, as the former is less expensive, does not require library preparation, and requires minimal starting material.

The ability to identify amplifications and deletions is of increasing importance in neuro-oncology. They can identify potential therapeutic targets and help distinguish different categories of brain cancers. For example, based on imaging and clinical findings, GBM and lymphoma can have overlapping presentations but face drastically different clinical approaches. Real-CSF has the potential to distinguish between these entities based on patterns of chromosomal alterations and apparent somatic mutations. GBM frequently has gains on 7p and 7q and losses on 10p and 10q—all infrequently observed in lymphoma. Conversely, lymphoma often has a gain on 18q and very few chromosome arm losses. These chromosomal alterations alone could accurately distinguish 73% of the Real-CSF-positive GBM and lymphomas in the current cohort. With additional samples, we anticipate that the performance will improve and will allow for accurate identification and classification of other cancer types beyond just those tested in the current study. For example, though we did not examine samples derived from individuals with oligodendroglioma, we anticipate being able to identify the canonical 1p/19q co-deletion.

The standard-of-care treatment for GBM includes concomitant chemo- and radiation therapy. Discerning true disease recurrence from treatment-related changes (pseudoprogression) on imaging can be very challenging.[34] Frequently, individuals are taken to surgery for pathological confirmation of disease status, and approximately 30% of cases are negative and only have treatment effect on histological examination.[35] If a biomarker was able to discern active disease from pseudoprogression, it may obviate the need for surgery in select patients. GLIA 566 is a case that suggests Real-CSF may be able to serve as such a biomarker. The subject had completed treatment with temozolomide and radiation therapy but was found to have progressive enhancement on MRI and was taken to surgery to distinguish tumor progression from treatment effect. Real-CSF accurately demonstrated active disease, which was confirmed

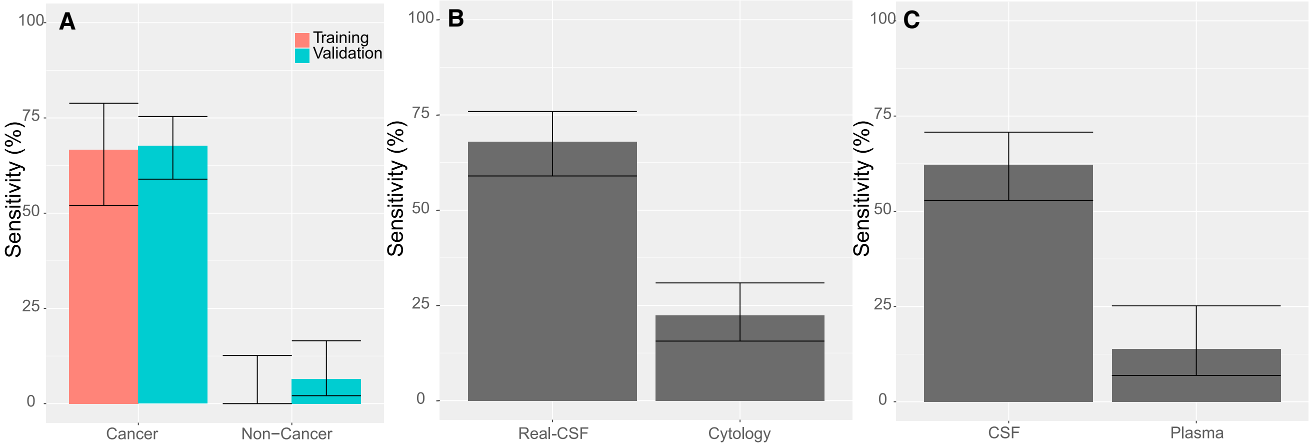

Figure 2. Evaluation of Real-CSF

(A) Comparison of performance of Real-CSF in the training and validation partitions. Medulloblastoma is not illustrated in this figure because it was not included in the training set.
(B) Comparison of performance of Real-CSF with cytology.
(C) Comparison of Real-CSF performance in CSF and plasma.
Error bars represent confidence intervals as calculated by the Wilson score interval.

upon pathological examination. The opposite was true for GLIA 543, where the individual was taken for surgical resection for suspected recurrence after chemotherapy and radiation therapy for GBM. Histopathological examination did not demonstrate active disease and was deemed to have pseudoprogression. Real-CSF was negative in this case and was able to accurately reflect what was seen upon pathological examination. In the future, testing with an assay such as Real-CSF may help accurately identify disease status without the need for neurosurgical intervention.

One of the strengths of our study is that it is the first to report a relatively large number (96) of CSF samples from patients without cancer but with a variety of other neurological conditions that can mimic cancer. These included patients with inflammatory, autoimmune, degenerative, congenital, and vascular conditions affecting the brain and CNS. Even so, the specificity in the validation set was 94%, comparable to CSF cytology.[2] While survival data were available for only a small fraction of subjects, our results suggest that individuals with positive CSF-tDNA levels have worse PFS and OS.

In summary, Real-CSF is a simple molecular assay that can assess the presence of aneuploidy using a single primer pair to aid in the management of patients with suspected cancers of the brain. We envision several clinical applications for Real-CSF use. For maximum sensitivity, Real-CSF could be combined with mutation or methylation markers while retaining specificity. Samples with limited input DNA, however, may not have sufficient material for mutation or methylation analysis let alone querying multiple analytes. Real-CSF works in ultra-low concentrations as little as a few pg and could aid in diagnosis when there is insufficient material for other analytes. In some clinical settings such as pediatric patients, Real-CSF could act as a stand-alone test given the relative paucity of somatic mutations in many brain cancers of childhood.[36,37]

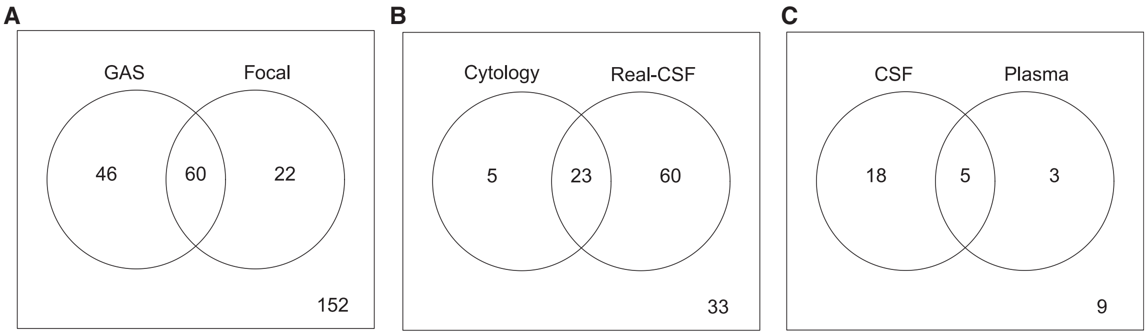

Figure 3. Venn diagrams

(A) Overlap of samples identified based on the global aneuploidy score (GAS) and panel of focal amplifications.
(B) Overlap of calls based on cytology and Real-CSF.
(C) Overlap of Real-CSF calls in matched CSF and plasma samples.

Limitations of the study

Our study has several limitations. Although the total number of samples is reasonably large compared with previously published CSF biomarker studies, confidence limits for subtypes of cancers could be narrowed by studying more patients. Second, we defined thresholds based on the training set and then used them to assess performance in an independent validation set derived from several different institutions. The optimal way to define thresholds for Real-CSF would be to use the training set to develop the model, use the validation set to define the thresholds, and then use a third, independent cohort (a "test set") to establish sensitivity and specificity. We did not have a sufficient number of samples to achieve this in the current study. However, the similarity in performance of the training and validation sets suggests that we will achieve similar sensitivities and specificities in independent cohorts in the future. A third limitation is that this study was retrospective in nature. A large, prospective study will be required to document that Real-CSF can become a useful diagnostic tool. The results reported here establish the conceptual and practical foundation for such a future study, which could have an impact on standard of care.

STAR★METHODS

Detailed methods are provided in the online version of this paper and include the following:

- KEY RESOURCES TABLE
- RESOURCE AVAILABILITY
  - Lead contact
  - Materials availability
  - Data and code availability
- EXPERIMENTAL MODEL AND SUBJECT DETAILS
  - Sample acquisition
- METHOD DETAILS
  - DNA purification
  - Real-CSF
  - Chromosome copy number alterations in CSF DNA
  - Chromosome copy number alterations in plasma cfDNA
  - Focal amplifications
  - WGS
- QUANTIFICATION AND STATISTICAL ANALYSIS

SUPPLEMENTAL INFORMATION

Supplemental information can be found online at https://doi.org/10.1016/j.xcrm.2023.101148.

ACKNOWLEDGMENTS

We would like to thank our patients for their bravery and willingness to participate in research studies. This work was supported by the Thomas M. Hohman Memorial Cancer Research Foundation. In addition, the work was supported by NIH grants RA37 CA230400, U01CA230691, and 5R21TR004059. This research was conducted using data or samples made available by The Children's Brain Tumor Network (formerly the Children's Brain Tumor Tissue Consortium). Grant support for this work was provided by the Burroughs Wellcome Career Award for Medical Scientists (C. Bettegowda); R21NS113016 (C. Bettegowda); RA37 CA230400 (C. Bettegowda); U01CA230691 (C. Bettegowda); Oncology Core CA 06973 (B.V., K.W.K., N.P.); Earlier Detection of Cancers Using Non-Plasma Liquid Biopsies (N.P., C. Bettegowda); The Virginia and D.K. Ludwig Fund for Cancer Research (B.V., K.W.K., N.P., C. Bettegowda, C.D.); the Thomas M. Hohman Memorial Cancer Research Foundation (C. Bettegowda); Alex's Lemonade Stand Foundation (C. Bettegowda); The Sol Goldman Sequencing Facility at Johns Hopkins (B.V.); The Conrad R. Hilton Foundation (N.P., B.V., K.W.K.); Benjamin Baker Endowment 80049589 and Ovarian Cancer SPORE DRP 80057309 (C.D.); and Swim Across America (C.D.).

AUTHOR CONTRIBUTIONS

Conceptualization, C.D. and C. Bettegowda; methodology, C.D., B.V., N.P., K.W.K., and C. Bettegowda; software, C.D., S.C., and M.S.; validation, C.D., S.C., M.S., B.V., N.P., K.W.K., and C. Bettegowda; formal analysis, C.D., S.C., M.S., T.D.A., J.R.-T., Y.W., A.M., B.A., J.D., J.M., D.R., S.N., D.B., K.T., L.D., M.P., J.P., N.N., N. Silliman, C. Blair, K.J., G.L.G., M.G., C.M.J., E.M.J., J.L., M.L., D.M., J.W., J.N., C.K., N. Smith, K.C.S., C.A.P., M.G., M.H., K.W.K., N.P., B.V., and C. Bettegowda; investigation, C.D., S.C., M.S., T.D.A., J.R.-T., Y.W., A.M., B.A., J.D., J.M., D.R., S.N., D.B., K.T., L.D., M.P., J.P., N.N., N. Silliman, C. Blair, K.J., G.L.G., M.G., C.M.J., E.M.J., J.L., M.L., D.M., J.W., J.N., C.K., N. Smith, K.C.S., C.A.P., M.G., M.H., K.W.K., N.P., B.V., and C. Bettegowda; resources, C.D., S.C., M.S., T.D.A., J.R.-T., Y.W., A.M., B.A., J.D., J.M., D.R., S.N., D.B., K.T., L.D., M.P., J.P., N.N., N. Silliman, C. Blair, K.J., G.L.G., M.G., C.M.J., E.M.J., J.L., M.L., D.M., J.W., J.N., C.K., N. Smith, K.C.S., C.A.P., M.G., M.H., K.W.K., N.P., B.V., and C. Bettegowda; data curation, C.D., S.C., M.S., T.D.A., J.R.-T., Y.W., A.M., B.A., J.D., J.M., D.R., S.N., D.B., K.T., L.D., M.P., J.P., N.N., N. Silliman, C. Blair, K.J., G.L.G., M.G., C.M.J., E.M.J., J.L., M.L., D.M., J.W., J.N., C.K., N. Smith, K.C.S., C.A.P., M.G., M.H., K.W.K., N.P., B.V., and C. Bettegowda; writing – original draft, C.D., B.V., N.P., K.W.K., and C. Bettegowda; writing – review & editing, C.D., B.V., N.P., K.W.K., and C. Bettegowda; visualization, C.D., B.V., N.P., K.W.K., and C. Bettegowda; supervision, C.D., B.V., N.P., K.W.K., and C. Bettegowda; project administration, C.D., B.V., N.P., K.W.K., and C. Bettegowda; funding acquisition, C.D., S.C., M.S., T.D.A., J.R.-T., Y.W., A.M., B.A., J.D., J.M., D.R., S.N., D.B., K.T., L.D., M.P., J.P., N.N., N. Silliman, C. Blair, K.J., G.L.G., M.G., C.M.J., E.M.J., J.L., M.L., D.M., J.W., J.N., C.K., N. Smith, K.C.S., C.A.P., M.G., M.H., K.W.K., N.P., B.V., and C. Bettegowda.

DECLARATION OF INTERESTS

B.V., K.W.K., and N.P. are founders of Thrive Earlier Detection, an Exact Sciences Company. K.W.K., N.P., and C.D. are consultants to Thrive Earlier Detection. B.V., K.W.K., N.P., and C.D. hold equity in Exact Sciences. B.V., K.W.K., and N.P. are founders of and own equity in ManaT Bio. K.W.K. and N.P. are consultants to and own equity in Haystack Oncology, Neophore, and Personal Genome Diagnostics. K.W.K., B.V., and N.P. hold equity in and are consultants to CAGE Pharma. B.V. is a consultant to and holds equity in Catalio Capital Management and may be a consultant to and hold equity in Haystack Oncology. B.V. owns equity in CAGE, Neophore, and Personal Genome Diagnostics. C. Bettegowda is a consultant to Depuy-Synthes, Bionaut Labs, Haystack Oncology, and Galectin Therapeutics. C. Bettegowda is a co-founder of OrisDx. C. Bettegowda and C.D. are co-founders of Belay Diagnostics. The companies named above, as well as other companies, have licensed previously described technologies related to the work described in this paper from Johns Hopkins University. B.V., K.W.K., N.P., C. Bettegowda, and C.D. are inventors on some of these technologies. Licenses to these technologies are or will be associated with equity or royalty payments to the inventors as well as to Johns Hopkins University. Patent applications on the work described in this paper may be filed by Johns Hopkins University. The terms of all these arrangements are being managed by Johns Hopkins University in accordance with its conflict of interest policies. M.H. is on the data safety monitoring board for Parexel and Advarra and has received an honorarium from Pfizer. K.S. received an honorarium from Springworks Therapeutics and receives research funding from Springworks Therapeutics. J.N. receives research funding from Merck, AstraZeneca, BMS, Amgen, Novartis, and Roche/Genetech and is a consulting/advisory board for Merck,

AstraZeneca, BMS, Amgen, Novartis, Roche/Genetech, Takeda, Pfizer, Daiichi Sankyo, and NGM Biosciences. C.J. is a co-founder with equity interest in Egret Therapeutics and receives research support from Biohaven.

Received: March 27, 2023
Revised: May 30, 2023
Accepted: July 13, 2023
Published: August 7, 2023

REFERENCES

1. Ostrom, Q.T., Patil, N., Cioffi, G., Waite, K., Kruchko, C., and Barnholtz-Sloan, J.S. (2020). CBTRUS Statistical Report: Primary Brain and Other Central Nervous System Tumors Diagnosed in the United States in 2013–2017. Neuro Oncol. *22*, iv1–iv96. https://doi.org/10.1093/neuonc/noaa200.
2. Weston, C.L., Glantz, M.J., and Connor, J.R. (2011). Detection of cancer cells in the cerebrospinal fluid: current methods and future directions. Fluids Barriers CNS *8*, 14. https://doi.org/10.1186/2045-8118-8-14.
3. Preusser, M., and Hainfellner, J.A. (2012). Chapter 11 - CSF and laboratory analysis (tumor markers). In Handbook of Clinical Neurology Neuro-Oncology, M.J. Aminoff, F. Boller, and D.F. Swaab, eds. (Elsevier), pp. 143–148. https://doi.org/10.1016/B978-0-444-52138-5.00011-6.
4. Van Mieghem, E., Wozniak, A., Geussens, Y., Menten, J., De Vleeschouwer, S., Van Calenbergh, F., Sciot, R., Van Gool, S., Bechter, O.E., Demaerel, P., et al. (2013). Defining pseudoprogression in glioblastoma multiforme. Eur. J. Neurol. *20*, 1335–1341. https://doi.org/10.1111/ene.12192.
5. Chen, C.-C., Hsu, P.-W., Erich Wu, T.-W., Lee, S.-T., Chang, C.-N., Wei, K.C., Chuang, C.-C., Wu, C.-T., Lui, T.-N., Hsu, Y.-H., et al. (2009). Stereotactic brain biopsy: Single center retrospective analysis of complications. Clin. Neurol. Neurosurg. *111*, 835–839. https://doi.org/10.1016/j.clineuro.2009.08.013.
6. Malone, H., Yang, J., Hershman, D.L., Wright, J.D., Bruce, J.N., and Neugut, A.I. (2015). Complications Following Stereotactic Needle Biopsy of Intracranial Tumors. World Neurosurg. *84*, 1084–1089. https://doi.org/10.1016/j.wneu.2015.05.025.
7. Nishihara, M., Sasayama, T., Kudo, H., and Kohmura, E. (2011). Morbidity of Stereotactic Biopsy for Intracranial Lesions. Kobe J. Med. Sci. *56*, E148–E153.
8. Pasternak, K.A., Schwake, M., Warneke, N., Masthoff, M., Zawy Alsofy, S., Suero Molina, E., Stummer, W., and Schipmann, S. (2021). Evaluation of 311 contemporary cases of stereotactic biopsies in patients with neoplastic and non-neoplastic lesions-diagnostic yield and management of non-diagnostic cases. Neurosurg. Rev. *44*, 2597–2609. https://doi.org/10.1007/s10143-020-01394-0.
9. Bettegowda, C., Sausen, M., Leary, R.J., Kinde, I., Wang, Y., Agrawal, N., Bartlett, B.R., Wang, H., Luber, B., Alani, R.M., et al. (2014). Detection of Circulating Tumor DNA in Early- and Late-Stage Human Malignancies. Sci. Transl. Med. *6*, 224ra24. https://doi.org/10.1126/scitranslmed.3007094.
10. De Mattos-Arruda, L., Mayor, R., Ng, C.K.Y., Weigelt, B., Martínez-Ricarte, F., Torrejon, D., Oliveira, M., Arias, A., Raventos, C., Tang, J., et al. (2015). Cerebrospinal fluid-derived circulating tumour DNA better represents the genomic alterations of brain tumours than plasma. Nat. Commun. *6*, 8839. https://doi.org/10.1038/ncomms9839.
11. CCGA Consortium; Liu, M.C., Oxnard, G.R., Klein, E.A., Swanton, C., Seiden, M.V., Liu, M.C., Oxnard, G.R., Klein, E.A., Smith, D., et al. (2020). Sensitive and specific multi-cancer detection and localization using methylation signatures in cell-free DNA. Ann. Oncol. *31*, 745–759. https://doi.org/10.1016/j.annonc.2020.02.011.
12. Fitzgerald, R.C., Antoniou, A.C., Fruk, L., and Rosenfeld, N. (2022). The future of early cancer detection. Nat. Med. *28*, 666–677. https://doi.org/10.1038/s41591-022-01746-x.
13. Gu, W., Rauschecker, A.M., Hsu, E., Zorn, K.C., Sucu, Y., Federman, S., Gopez, A., Arevalo, S., Sample, H.A., Talevich, E., et al. (2021). Detection of Neoplasms by Metagenomic Next-Generation Sequencing of Cerebrospinal Fluid. JAMA Neurol. *78*, 1355–1366. https://doi.org/10.1001/jamaneurol.2021.3088.
14. Mouliere, F., Mair, R., Chandrananda, D., Marass, F., Smith, C.G., Su, J., Morris, J., Watts, C., Brindle, K.M., and Rosenfeld, N. (2018). Detection of cell-free DNA fragmentation and copy number alterations in cerebrospinal fluid from glioma patients. EMBO Mol. Med. *10*, e9323. https://doi.org/10.15252/emmm.201809323.
15. Figueroa, J.M., and Carter, B.S. (2018). Detection of glioblastoma in biofluids. J. Neurosurg. *129*, 334–340. https://doi.org/10.3171/2017.3.JNS162280.
16. Boire, A., Brandsma, D., Brastianos, P.K., Le Rhun, E., Ahluwalia, M., Junck, L., Glantz, M., Groves, M.D., Lee, E.Q., Lin, N., et al. (2019). Liquid biopsy in central nervous system metastases: a RANO review and proposals for clinical applications. Neuro Oncol. *21*, 571–584. https://doi.org/10.1093/neuonc/noz012.
17. Soffietti, R., Bettegowda, C., Mellinghoff, I.K., Warren, K.E., Ahluwalia, M.S., De Groot, J.F., Galanis, E., Gilbert, M.R., Jaeckle, K.A., Le Rhun, E., et al. (2022). Liquid biopsy in gliomas: A RANO review and proposals for clinical applications. Neuro Oncol. *24*, 855–871. https://doi.org/10.1093/neuonc/noac004.
18. Mattox, A.K., Yan, H., and Bettegowda, C. (2019). The potential of cerebrospinal fluid–based liquid biopsy approaches in CNS tumors. Neuro Oncol. *21*, 1509–1518. https://doi.org/10.1093/neuonc/noz156.
19. Orzan, F., De Bacco, F., Lazzarini, E., Crisafulli, G., Gasparini, A., Dipasquale, A., Barault, L., Macagno, M., Persico, P., Pessina, F., et al. (2023). Liquid Biopsy of Cerebrospinal Fluid Enables Selective Profiling of Glioma Molecular Subtypes at First Clinical Presentation. Clin. Cancer Res. *29*, 1252–1266. https://doi.org/10.1158/1078-0432.CCR-22-2903.
20. Rahimi, J., and Woehrer, A. (2018). Chapter 35 - Overview of cerebrospinal fluid cytology. In Handbook of Clinical Neurology Neuropathology, G.G. Kovacs and I. Alafuzoff, eds. (Elsevier), pp. 563–571. https://doi.org/10.1016/B978-0-12-802395-2.00035-3.
21. Morell, A.A., Shah, A.H., Cavallo, C., Eichberg, D.G., Sarkiss, C.A., Benveniste, R., Ivan, M.E., and Komotar, R.J. (2019). Diagnosis of primary central nervous system lymphoma: a systematic review of the utility of CSF screening and the role of early brain biopsy. Neurooncol. Pract. *6*, 415–423. https://doi.org/10.1093/nop/npz015.
22. Hoang-Xuan, K., Bessell, E., Bromberg, J., Hottinger, A.F., Preusser, M., Rudà, R., Schlegel, U., Siegal, T., Soussain, C., Abacioglu, U., et al. (2015). Diagnosis and treatment of primary CNS lymphoma in immunocompetent patients: guidelines from the European Association for Neuro-Oncology. Lancet Oncol. *16*, e322–e332. https://doi.org/10.1016/S1470-2045(15)00076-5.
23. Naidoo, J., Schreck, K.C., Fu, W., Hu, C., Carvajal-Gonzalez, A., Connolly, R.M., Santa-Maria, C.A., Lipson, E.J., Holdhoff, M., Forde, P.M., et al. (2021). Pembrolizumab for patients with leptomeningeal metastasis from solid tumors: efficacy, safety, and cerebrospinal fluid biomarkers. J. Immunother. Cancer *9*, e002473. https://doi.org/10.1136/jitc-2021-002473.
24. Knouse, K.A., Davoli, T., Elledge, S.J., and Amon, A. (2017). Aneuploidy in Cancer: Seq-ing Answers to Old Questions. Annu. Rev. Cancer Biol. *1*, 335–354. https://doi.org/10.1146/annurev-cancerbio-042616-072231.
25. Gröbner, S.N., Worst, B.C., Weischenfeldt, J., Buchhalter, I., Kleinheinz, K., Rudneva, V.A., Johann, P.D., Balasubramanian, G.P., Segura-Wang, M., Brabetz, S., et al. (2018). The landscape of genomic alterations across childhood cancers. Nature *555*, 321–327. https://doi.org/10.1038/nature25480.
26. Bočkaj, I., Martini, T.E.I., Smit, M.J., Armandari, I., Bakker, B., Wardenaar, R., Meeuwsen-de Boer, T.G.J., Bakker, P.L., Spierings, D.C.J., Hoving, E.W., et al. (2022). Chromosomal Instability Characterizes Pediatric

Medulloblastoma but Is Not Tolerated in the Developing Cerebellum. Int. J. Mol. Sci. *23*, 9852. https://doi.org/10.3390/ijms23179852.

27. Douville, C., Cohen, J.D., Ptak, J., Popoli, M., Schaefer, J., Silliman, N., Dobbyn, L., Schoen, R.E., Tie, J., Gibbs, P., et al. (2020). Assessing aneuploidy with repetitive element sequencing. Proc. Natl. Acad. Sci. *117*, 4858–4863. https://doi.org/10.1073/pnas.1910041117.
28. Douville, C., Springer, S., Kinde, I., Cohen, J.D., Hruban, R.H., Lennon, A.M., Papadopoulos, N., Kinzler, K.W., Vogelstein, B., and Karchin, R. (2018). Detection of aneuploidy in patients with cancer through amplification of long interspersed nucleotide elements (LINEs). Proc. Natl. Acad. Sci. *115*, 1871–1876. https://doi.org/10.1073/pnas.1717846115.
29. FireBrowse http://firebrowse.org/#.
30. Wan, N., Weinberg, D., Liu, T.-Y., Niehaus, K., Ariazi, E.A., Delubac, D., Kannan, A., White, B., Bailey, M., Bertin, M., et al. (2019). Machine learning enables detection of early-stage colorectal cancer by whole-genome sequencing of plasma cell-free DNA. BMC Cancer *19*, 832. https://doi.org/10.1186/s12885-019-6003-8.
31. Alzial, G., Renoult, O., Paris, F., Gratas, C., Clavreul, A., and Pecqueur, C. (2022). Wild-type isocitrate dehydrogenase under the spotlight in glioblastoma. Oncogene *41*, 613–621. https://doi.org/10.1038/s41388-021-02056-1.
32. Raman, L., Dheedene, A., De Smet, M., Van Dorpe, J., and Menten, B. (2019). WisecondorX: improved copy number detection for routine shallow whole-genome sequencing. Nucleic Acids Res. *47*, 1605–1614.
33. Liu, A.P.Y., Smith, K.S., Kumar, R., Paul, L., Bihannic, L., Lin, T., Maass, K.K., Pajtler, K.W., Chintagumpala, M., Su, J.M., et al. (2021). Serial assessment of measurable residual disease in medulloblastoma liquid biopsies. Cancer Cell *39*, 1519–1530.e4. https://doi.org/10.1016/j.ccell.2021.09.012.
34. Abbasi, A.W., Westerlaan, H.E., Holtman, G.A., Aden, K.M., van Laar, P.J., and van der Hoorn, A. (2018). Incidence of Tumour Progression and Pseudoprogression in High-Grade Gliomas: a Systematic Review and Meta-Analysis. Clin. Neuroradiol. *28*, 401–411. https://doi.org/10.1007/s00062-017-0584-x.
35. Woodworth, G.F., Garzon-Muvdi, T., Ye, X., Blakeley, J.O., Weingart, J.D., and Burger, P.C. (2013). Histopathological correlates with survival in reoperated glioblastomas. J. Neuro Oncol. *113*, 485–493. https://doi.org/10.1007/s11060-013-1141-3.
36. Northcott, P.A., Buchhalter, I., Morrissy, A.S., Hovestadt, V., Weischenfeldt, J., Ehrenberger, T., Gröbner, S., Segura-Wang, M., Zichner, T., Rudneva, V.A., et al. (2017). The whole-genome landscape of medulloblastoma subtypes. Nature *547*, 311–317. https://doi.org/10.1038/nature22973.
37. Pajtler, K.W., Witt, H., Sill, M., Jones, D.T.W., Hovestadt, V., Kratochwil, F., Wani, K., Tatevossian, R., Punchihewa, C., Johann, P., et al. (2015). Molecular Classification of Ependymal Tumors across All CNS Compartments, Histopathological Grades, and Age Groups. Cancer Cell *27*, 728–743. https://doi.org/10.1016/j.ccell.2015.04.002.
38. Olshen, A.B., Venkatraman, E.S., Lucito, R., and Wigler, M. (2004). Circular binary segmentation for the analysis of array-based DNA copy number data. Biostatistics *5*, 557–572. https://doi.org/10.1093/biostatistics/kxh008.
39. Wang, Y., Douville, C., Cohen, J.D., Mattox, A., Curtis, S., Silliman, N., Popoli, M., Ptak, J., Dobbyn, L., Nehme, N., et al. (2022). Detection of Rare Mutations, Copy Number Variation, and DNA Methylation in the Same Template DNA Molecules. https://doi.org/10.1101/2022.12.06.22283116.

CellPress
OPEN ACCESS

STAR★METHODS

KEY RESOURCES TABLE

REAGENT or RESOURCE	SOURCE	IDENTIFIER
**Oligonucleotides**		
DNA purification	Biochain	catalog #K5011625MA
Oligonucleotides (Forward Primer)	IDT	cgacgtaaaacgacggccagtNNNNNNNNNNNNNNNNGGTGAA ACCCCGTCTCTACA
Oligonucleotides (Reverse Primer)	IDT	cacacaggaaacagctatgaccatgCCTCCTAAGTAGCTGGGAC TACAG
**Other**		
Beads	Beckman	# a63880
NEBNext Ultra II Q5 Master Mix	New England Biolabs	#M0544S
**Deposited data**		
Sequencing Data	This Paper	European Genome Archive Study ID: EGAS00001007401
**Software and algorithms**		
Bioinformatics Scripts	This Paper	Zenodo: https://zenodo.org/record/3656943#.YaZZCdDMKUk

RESOURCE AVAILABILITY

Lead contact

Further information and requests for resources and reagents should be directed to and will be fulfilled by the lead contact, Chetan Bettegowda (Cbetteg1@jhmi.edu).

Materials availability

This study did not generate new unique reagents.

Data and code availability

- De-identified raw data have been deposited at the European Genome Archive using Study ID: EGAS00001007401.
- The bioinformatic pipeline is available at https://zenodo.org/record/3656943#.YaZZCdDMKUk.
- Any additional information required to reanalyze the data reported in this work paper is available from the lead contact upon request.

EXPERIMENTAL MODEL AND SUBJECT DETAILS

Sample acquisition

This study was cross-sectional in design. Patients were recruited as part of an Institutional Review Board-approved, multi-institutional study to develop biomarkers for central nervous system tumors using cerebrospinal fluid. The four institutions involved (Johns Hopkins, University of Michigan, Penn State University, Children Tissue Brain Tumor Tissue Consortium (CBTTC)) are tertiary centers that care for patients referred for management of central nervous system tumors. In general, patients underwent sampling on the day of enrollment and only tumors with radiographic confirmation with contrast enhanced MRI were included in the study. Radiographic findings of disease were based on the findings of a board certified-neuroradiologist at each site. In total there were 280 samples collected for this study. Pathologic diagnosis for all cases was verified by board-certified neuropathologists at the site of enrollment. Patients whose diagnosis was GBM, CNS lymphoma, medulloblastoma or metastasis from outside the brain comprised the true positive subset. Patients who were not diagnosed with any neoplastic disease comprised the true negative set. We were able to assess plasma samples from 65 cancer patients of which 35 had matched CSF for comparison purposes.

Samples were pre-specified into training and validation cohorts based simply on the time at which the sample became available for evaluation in our laboratory at Johns Hopkins. An initial batch of samples from Johns Hopkins were labeled as training samples. To reduce potential cohort biases and overfitting from machine learning, all samples from the Penn State University, CBTTC, and the University of Michigan were labeled as validation samples. The remaining Johns Hopkins samples not evaluated in the initial batch of samples were included in the validation set.

METHOD DETAILS

DNA purification

CSF was frozen in its entirety at -80°C until DNA purification, and the entire volume of CSF (cells plus fluid) was used for DNA purification. The amount of CSF used for purification ranged from 0.5 to 1 mL. CSF using Biochain reagents according to the manufacturer's instructions (catalog #K5011625MA). Note that the K5011625MA workflow is designed for plasma, but we used it both for purifying CSF and for purifying plasma.

Real-CSF

A single primer pair was used to amplify ~350,000 short interspersed nuclear elements (SINEs) spread throughout the genome.[27] PCR was performed in 25 uL reactions containing 7.25 uL of water, 0.125 uL of each primer, 12.5 uL of NEBNext Ultra II Q5 Master Mix (New England Biolabs cat # M0544S), and 5 uL of DNA. The cycling conditions were: one cycle of 98°C for 120 s, then 15 cycles of 98°C for 10 s, 57°C for 120 s, and 72°C for120 s. Each sample was assessed in eight independent reactions, and the amount of DNA per reaction varied from ~0.1 ng to 0.25 ng. A second round of PCR was then performed to add dual indexes (barcodes) to each PCR product prior to sequencing. The second round of PCR was performed in 25 uL reactions containing 7.25 uL of water, 0.125 uL of each primer, 12.5 uL of NEBNext Ultra II Q5 Master Mix (New England Biolabs cat # M0544S), and 5 uL of DNA containing 5% of the PCR product from the first round. The cycling conditions were: one cycle of 98°C for 120 s, then 15 cycles of 98°C for 10 s, 65°C for 15 s, and 72°C for 120 s. Amplification products from the second round were purified with AMPure XP beads (Beckman cat # a63880), as per the manufacturer's instructions, prior to sequencing.

Sequencing was performed on an Illumina HiSeq 4000. The sequencing reads from the 8 replicates of each sample were summed for bioinformatic analysis. The average number of the summed, uniquely aligned reads was 10.5 million (interquartile range, 8.0-12.7 million). The bioinformatic methods and pipeline used to process the raw sequencing data are available at (https://zenodo.org/record/3656943#.YaZZCdDMKUk).

Chromosome copy number alterations in CSF DNA

Copy number alterations for CSF samples were calculated using the Within Sample Aneuploidy Detection Algorithm (WALDO).[28] For this analysis, we generated a reference panel from 15 non-cancer CSF samples. For all samples in the reference panel, read depths were aggregated into 5,344 non-overlapping autosomal 500-kb intervals. The depths were normalized to account for coverage differences. PCA Normalization was performed for the euploid reference panel for all 5,344 500kb intervals. This type of normalization attempts to mitigate the impact of highly correlated regions and limit potential technical artifacts. A full protocol of this normalization is detailed in Douville et al. 2020.[27]

After generating a reference panel, copy number analysis could be performed on test samples. For each test sample, read depths were aggregated across the 5,344 non-overlapping autosomal 500-kb intervals. PCA normalization was performed. Chromosome arms were segmented using the circular binary segmentation algorithm (CBS).[38] Germline copy number variations and outlier intervals were excluded and statistical significance was determined across the entire arm. This procedure was performed for all 39 non-acrocentric chromosome arms. The test sample's 39 chromosome arms were evaluated using a previously built supervised machine learning algorithm. This model generates a Global Aneuploidy Score (GAS) to discriminate between aneuploid and euploid samples. The predictive features of the model are the 39 chromosome arms (Z_w). The training examples were 3,999 previously published plasma samples. The negative class of 1348 presumably euploid samples were taken from individuals without cancer. The positive class was taken from 2651 aneuploid samples across 8 different cancer types. We specifically built a support vector machine (SVM) and trained the model with the e1071 package in R, using a radial basis kernel and default parameters. The full bioinformatic pipeline is available at https://zenodo.org/record/3656943#.YaZZCdDMKUk.

Chromosome copy number alterations in plasma cfDNA

To identify copy number alterations in plasma we repeated the steps from above but made one key change. We reconstructed the euploid reference panel using a set of 1,500 euploid plasma samples. We then repeated the same protocol as above to calculate the statistical significances for each arm and generate Global Aneuploidy Scores.

Focal amplifications

RealSeqS amplicons overlapping the genomic coordinates of the gene of interest, plus 1 Mb on either side of the gene, were identified. The summed read counts ($Observed_{gene}$) across these amplicons were then determined for each sample. We estimated the expected read depth for a particular gene of interest using our reference panel (μ_{gene}).

For each test sample, we calculated the total autosomal sequencing depth (Coverage). We multiplied (μ_{gene}) which was calculated from the reference panel by the observed coverage in our test sample to estimate the expected number of reads across the gene of interest (λ_{gene}) for the given coverage. We assumed that the count data followed a Poisson distribution. Then, we aggregate the read depth across the gene of interest ($Observed_{gene}$) for this test sample. We calculated the statistical significance for each gene of interest using the following equation.

$$Z_{gene} = \frac{Observed_{gene} - \lambda_{gene}}{\sqrt{\lambda_{gene}}}$$

This protocol was followed for both CSF and plasma samples. The only difference between CSF and plasma was the euploid reference panel used to generate the expected depth for each gene, as noted above. Scores for both CSF and plasma are detailed in Table S2.

WGS

We developed a custom library preparation workflow that can efficiently recover input DNA fragments and simultaneously incorporate double-stranded molecular barcodes. Conceptual and practical details of this strategy are discussed in Wang et al.[39] In brief, libraries were prepared with both cell-free DNA and genomic DNA in the CSF using an Accel-NGS 2S DNA Library Kit (Swift Biosciences, 21024) with the following critical modifications: (1) DNA was pretreated with 3 U of USER enzyme (New England BioLabs, M5505L) for 15 min at 37°C to excise uracil bases; (2) the SPRI bead/PEG NaCl ratios used after each reaction were 2.0×, 1.8×, 1.2× and 1.05× for end repair 1, end repair 2, ligation 1 and ligation 2, respectively; (3) a custom 50 μM 3′ adapter was substituted for reagent Y2 and a custom 42 μM 5′ adapter was substituted for reagent B2. Libraries were subsequently PCR amplified in 50-μl reactions using primers targeting the ligated adapters. The following reaction conditions were used: 1× NEBNext Ultra II Q5 Master Mix (New England BioLabs, M0544L), 2 μM universal forward primer and 2 μM universal reverse primer. Libraries were amplified with 5, 7 or 11 cycles of PCR, depending on how many experiments were planned, according to the following protocol: 98°C for 30 s, cycles of 98°C for 10 s, 65°C for 75 s and hold at 4°C. If five or seven cycles were used, the libraries were amplified in single 50-μl reactions. If 11 cycles were used, the libraries were divided into eight aliquots and amplified in eight 50-μl reactions, each supplemented with an additional 0.5 U of Q5 Hot Start High-Fidelity DNA Polymerase (New England BioLabs, M0493L), 1 μl of 10 mM dNTPs (New England BioLabs, N0447L) and 0.4 μl of 25 mM MgCl2 solution (New England BioLabs, B9021S). The products were purified with 1.8× SPRI beads (Beckman Coulter, B23317) and eluted in EB buffer (Qiagen). An average of 34.3 M unique reads pairs per sample (IQR 29.2M to 38.8M were obtained. Copy number alterations were identified with WisecondorX using 500kb intervals and default parameters.

QUANTIFICATION AND STATISTICAL ANALYSIS

Performance comparisons between the training and validation sets were assessed with the *Z* Score for 2 Population Proportions. The survival statistics were assessed using the log rank test.

Cell Reports Medicine, Volume *4*

Supplemental information

Seq-ing the SINEs of central nervous system tumors in cerebrospinal fluid

Christopher Douville, Samuel Curtis, Mahmoud Summers, Tej D. Azad, Jordina Rincon-Torroella, Yuxuan Wang, Austin Mattox, Bracha Avigdor, Jonathan Dudley, Joshua Materi, Divyaansh Raj, Sumil Nair, Debarati Bhanja, Kyle Tuohy, Lisa Dobbyn, Maria Popoli, Janine Ptak, Nadine Nehme, Natalie Silliman, Cherie Blair, Kathy Judge, Gary L. Gallia, Mari Groves, Christopher M. Jackson, Eric M. Jackson, John Laterra, Michael Lim, Debraj Mukherjee, Jon Weingart, Jarushka Naidoo, Carl Koschmann, Natalya Smith, Karisa C. Schreck, Carlos A. Pardo, Michael Glantz, Matthias Holdhoff, Kenneth W. Kinzler, Nickolas Papadopoulos, Bert Vogelstein, and Chetan Bettegowda

Supplemental Information:

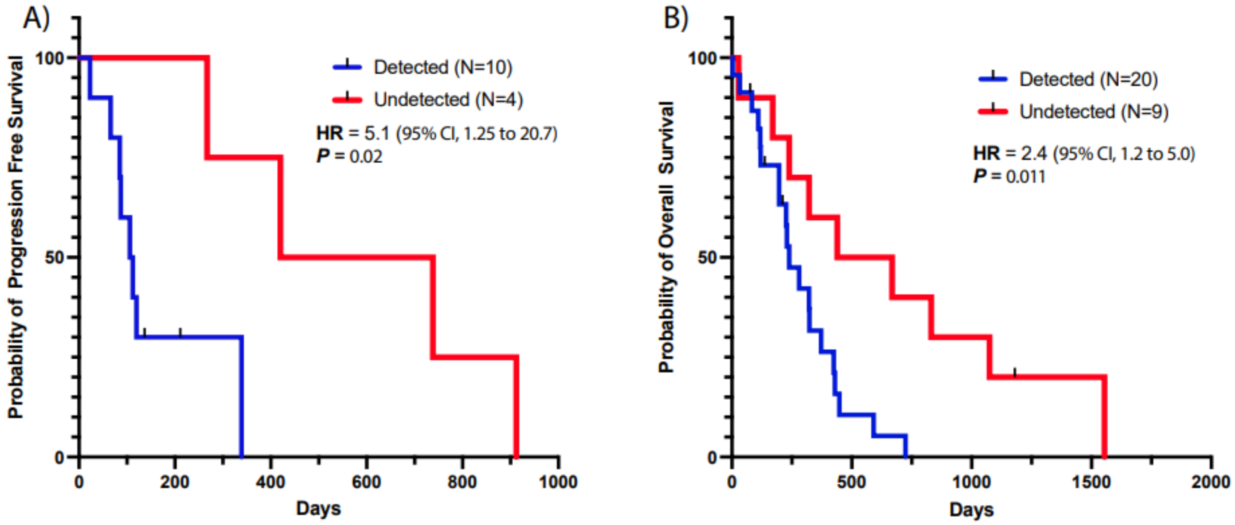

Supplemental Figure S1: Figure S1: Survival Analysis of new diagnosed GBM patients based on detectable copy number alterations in the CSF, related to STAR Methods. A) Progression Free Survival (PFS) B) Overall Survival (OS).

Supplemental Table S1: A Full Description of the Sample Demographics in the study, related to STAR Methods.

Supplemental Table S2: Genomic Coordinates for the Focal Regions Evaluated, related to Figure 1.

Supplemental Table S3: Real-CSF Raw Predictive Features Listed for all Manuscript Samples, related to STAR Methods.

Supplemental Table S4: Real-CSF Calls For Matched Plasma Samples, related to STAR Methods.
